# Migration and Aging in the Right Place: Older Puerto Rican Adults’ Narratives

**DOI:** 10.1093/geroni/igab046.1961

**Published:** 2021-12-17

**Authors:** Brooke Jespersen

**Affiliations:** Case Western Reserve University, Cleveland, Ohio, United States

## Abstract

This research examines meanings of aging in the “right” place (Golant, 2015) among older adults who have grown up and grown older in migratory contexts. This qualitative research is based on semi-structured and life history interviews with 30 low socio-economic status Puerto Rican adults over the age of 60 who reside in Cuyahoga County, Ohio and have engaged in Puerto Rico-US migration throughout the life course. Inductive thematic analysis of interviews revealed fraught, multi-scalar narratives of aging in the “right” place. At the level of residence type, older adults’ narratives exhibited a tension between interdependence and independence. That is to say, they struggled to reconcile cultural preferences for family-based living arrangements with fears of becoming a burden. At the level of nation, a similar tension manifested. Older adults reported navigating differential citizenship rights, access to healthcare and social services, natural disasters, and experiences of social inclusion and exclusion via migration between Puerto Rico and the US mainland. Thus, aging in the “right” place was complex, if not altogether elusive, as inequitable circumstances obliged older adults to make tradeoffs regardless of where they lived. These findings extend scholarship on aging in the “right” place, which has focused on residence type, by considering how older adults negotiate aging within and across households, communities, and nations. Moreover, these findings highlight how challenging aging in the “right” place can be for migrating and disadvantaged populations.

